# Hot Deformation and Microstructure Evolution of Metallic Materials

**DOI:** 10.3390/ma16041602

**Published:** 2023-02-14

**Authors:** Ivo Schindler

**Affiliations:** Faculty of Materials Science and Technology, VŠB – Technical University of Ostrava, 17. listopadu 2172/15, 70800 Ostrava, Czech Republic; ivo.schindler@vsb.cz

Hot plastic deformation is a key method of processing metallic materials and controlling their final properties through structure-forming processes. The efficiency of the bulk forming processes contributes significantly to the environmental aspects of our civilization’s sustainable development. The ability to exploit the structural potentiality of both traditional alloys and new, progressive materials is crucial in terms of economic growth.

Thus, the content of this Special Issue, “Hot Deformation and Microstructure Evolution of Metallic Materials”, focused not only on common technologies (e.g., rolling or forging), but also on various types of complex thermomechanical processing. The strong physical basis of the research work, the application of advanced hot deformation simulators and structural analysis methods, and the optimization computer simulations of forming processes were emphasized. Much attention was paid to the structure-forming processes, which are connected with the bulk forming and controlled cooling of various types of metallic materials (steels, alloys of aluminum including composites, magnesium, titanium, copper, etc.). It is interesting that the experiments describe in almost half of the articles were based on the application of a compression test—either uniaxial or with plane strain.

Many authors devoted themselves to the analysis of flow stress curves and their use in the creation of constitutive models; the description of the dynamic recrystallization kinetics using the calculated value of the activation energy at hot forming; the construction of processing maps, etc. Łukaszek-Sołek et al. [1] focused on the hot deformation behavior of 4130 steel and the optimization of its processing parameters. The flow stress curves obtained during compression tests, as well as the processing maps which elaborated on the basis of various flow stability criteria, were discussed. Processing parameters developed according to Prasad’s and Murty’s criteria were recommended for designing the technology for forging the investigated steel. Such parameters ensure the homogeneity and stability of the material flow, which was confirmed by the successful forging of 4130 steel in industrial conditions. Chen et al. [2] developed a flow stress model of 300 M steel using hot uniaxial tensile tests. Compared with uniaxial compression, the tensile flow stress was 29.1% higher because dynamic recrystallization softening was less sufficient in the tensile stress state. To eliminate the influence of sample necking on the stress–strain relationship, both the stress and the strain were calibrated using the cross-sectional area of the neck zone. A constitutive model was established based on the modified Arrhenius model, in which the individual parameters were described as a functions of strain. Hot deformation behavior of selected non-alloyed carbon steels was investigated by Kawulok et al. [3]. Based on the analysis of experimentally determined flow stress curves, material constants suitable for predicting the peak flow stress, σp; peak strain, εp; and critical strain, εcrDRX, necessary to induce dynamic recrystallization were determined. The validity of the predicted critical strains, εcrDRX, was then experimentally verified. Equations describing a simple linear dependence of the critical strain εcrDRX on the peak strain εp were derived for all investigated steels. The determined hot deformation activation energy Q decreased with increasing carbon content. Individual flow stress curves of the studied steels were mathematically described using the Cingara and McQueen model. Kittner, Ullmann, and Ulrich [4] performed a comparative study on the hot deformation behavior of as-cast and twin-roll cast (TRC) Mg-6.8Y-2.5Zn-0.4Zr alloy. TRC resulted in a finer microstructure. In the conventional as-cast state, dynamic recrystallization (DRX) was delayed by the coarse, blocky, long-period stacking-order phases. Optimum deformation conditions for both states are temperatures from 500 °C to 520 °C, and strain rates ranging from 0.01 s^−1^ to 0.1 s^−1^ for the as-cast material as well as a strain rate of 1 s^−1^ for the TRC material. Wang et al. [5] proposed the cold radial forging method and semi-solid isothermal treatment in the semi-solid isothermal compression (SSIC) process to fabricate high-quality, semi-solid billets of 6063 aluminum alloy. Constitutive equations were established based on the experimental data to predict the flow stress. Four stages (i.e., sharp increase, decrease, steady state, and slow increase) were observed in the true stress–true strain curve. The deformation mechanism for SSIC of cold radial forged alloy 6063 mainly included four modes: the liquid phase flow, grain slide or grain rotation along with the liquid film, slide among solid grains, and the plastic deformation of solid grains. The strain compensation model and processing maps to describe the hot deformation behavior of a metastable β titanium alloy was developed by Lypchanskyi et al. [6] at near and above β transus temperatures. The strain-compensated constitutive model was developed using the Arrhenius-type equation. The dynamic material modeling in combination with Prasad’s stability criterion made it possible to generate processing maps for the investigated deformation conditions. The high material flow stability under investigated deformation conditions was revealed. It was found that dynamic recovery was the main mechanism operating during the deformation of the investigated alloy. Zhang et al. [7] studied the DRX behavior of 47Zr-45Ti-5Al-3V alloy by using an experiment and numerical simulation method based on DEFORM-3D software and cellular automata (CA) over a range of deformation temperatures (850 to 1050 °C) and strain rates (10^−3^ to 100 s^−1^). With the rising deformation temperature and decreasing strain rate, the grain size (dDRX) and volume fraction (XDRX) of DRX were dramatically boosted. According to the developed kinetics models, the distributions of the dDRX and XDRX for DRX grains were predicted by DEFORM-3D. DRX microstructure evolution is simulated by CA. The nucleation and growth of DRX grains in the tested alloy during hot working can be simulated more accurately by CA simulation compared with finite element analysis. In research presented by Opěla et al. [8], conventional hot processing maps superimposed over flow stress maps or activation energy maps were utilized to study the correlations among the efficiency of power dissipation, flow stress, and activation energy evolution in the case of Cr-Mo low-alloyed steel. All maps were assembled on the basis of two flow curve datasets. The experimental set is the result of a series of uniaxial hot compression tests. The predicted flow curve dataset was calculated on the basis of the subsequent approximation procedure via a well-adapted artificial neural network. It was found that both flow stress and activation energy evolution were able to express changes in the studied steel caused by the hot compression deformation. A direct association with the course of power dissipation efficiency is evident. It can be stated that the activation energy processing maps represent another tool for finding the appropriate forming conditions and can be utilized as a support feature for the conventionally-used processing maps to extend their informative ability.

The authors of a total of five papers focused on the study and modeling of microstructure development during hot rolling and subsequent cooling. The grain size models of Hanoglu and Šarler [9] were one-way coupled to the macro-scale calculations performed with the slice model assumption. The macroscale solution was based on a novel radial basis function collocation method. This numerical method was truly meshless, as it involved space discretization in arbitrarily distributed nodes without meshing. Austenite grain size at each rolling pass, as well as the ferrite grain size at the end of rolling, were predicted in this simulation. It was also shown that based on the rolling schedule, it was highly likely that recrystallization would take place at each pass throughout a continuous rolling mill. The simulation system was coded as a user-friendly computer application for industrial use and ran on regular personal computers. The computational time for a typical rolling simulation is usually less than one hour. Lin et al. [10] studied a 6-pass continuous hot-rolling process followed by air cooling by means of a coupled multi-scale simulation approach. The finite element method was utilized to obtain macroscale thermomechanical parameters. The microstructure evolution during the recrystallization and austenite (γ)-to-ferrite (α) transformation was simulated by a mesoscale cellular automaton model. The driving force for α-phase nucleation and growth involved the contribution of the deformation stored energy inherited from hot-rolling. A detailed analysis demonstrated how the parameters, including strain rate, grain size, temperature, and inter-pass time, influenced the different mechanisms of recrystallization. Grain refinement induced by recrystallization and the γ→α phase transformation was also quantified. The simulated final α-fraction and the average α-grain size agreed reasonably well with the experimental microstructure. The kinetics of dynamic, meta-dynamic, and static recrystallization in high-carbon bainitic steel during hot deformation were described by Dembiczak and Knapiński [11]. The developed mathematical model takes into account the dependence of the changing kinetics on the structural size of the preliminary austenite grains, as well as the value of strain, strain rate, temperature, and time. Physical simulations were carried out by plain strain compression tests (PSCT). Based on dilatometric studies, growth of the austenite grain occurred under isothermal annealing conditions. The developed mathematical models were verified by a semi-industrial hot-rolling process. Sauer et al. [12] analyzed the microstructure development of Nb-microalloyed steel during rolling on a heavy-section mill. A modified microstructure evolution model was presented that better accounted for the influence of strain-induced precipitation (SIP) on the kinetics of static recrystallization. The model was verified using the plain strain compression simulations of rolling a round bar 100 mm in diameter. For indirect comparison with the model outputs, the SIP initiation time was determined based on the NbX precipitate size distribution obtained by transmission electron microscopy. Using the PSCT and the outputs from the microstructure evolution model, it was found that during conventional rolling, strain-induced precipitation occurred after the last pass and, thus, did not affect the austenite grain size. By lowering the rolling temperature, it was possible to reduce the grain size by up to 56 μm. Schindler et al. [13] studied the combined effect of austenitization temperature and pre-deformation on continuous cooling transformation (CCT) diagrams of 23MnNiCrMo5-3 steel. Based on the dilatometric tests and metallographic analyses, a total of five different diagrams were constructed and compared. Pre-deformation corresponding to the true strain of 0.35 or even 1.0 had no clear effect on the austenite decomposition kinetics at a deformation temperature of 880 °C. During the long-lasting cooling, recrystallization and, likely, coarsening of the new austenitic grains occurred, which almost eliminated the influence of pre-deformation on the temperatures of the diffusion-controlled phase transformations. Decreasing the deformation temperature to 830 °C led to a significant acceleration of the austenite→ferrite and austenite→pearlite transformations due to the applied strain of 1.0 only in the region of the cooling rate, between 3 and 35 °C·s^−1^. Acceleration of the diffusion-controlled phase transformations resulted from the formation of an austenitic microstructure with a mean grain size of approximately 4 µm. As the analysis of the stress–strain curves showed, grain refinement was carried out by dynamic and metadynamic recrystallization. At low cooling rates, the effect of plastic deformation on the kinetics of phase transformations was indistinct.

The other three articles also discuss the microstructure evolution. Zurutuza et al. [14] studied the effect of DRX in ultra-high strength boron-microalloyed steels optionally alloyed with niobium and molybdenum. Multi-pass torsion tests were performed to simulate plate rolling conditions, followed by direct quenching. For Nb-microalloyed steel, partial DRX occurred and resulted in local clusters of fine-sized equiaxed grains being dispersed within the pancaked austenitic structure. A recrystallized austenite fraction appeared and transformed into softer phase constituents after direct quenching. The addition of molybdenum suppressed DRX and ensured the formation of fully martensitic microstructures. Cojocaru et al. [15] analyzed the hot deformation behavior of UNS S32750 Super-Duplex Stainless Steel during processing by upsetting. The resulting samples were examined by electron backscatter diffraction (EBSD) to establish the evolution of the phases present in the structure from several points of view: nature, distribution, morphology (size and shape), and their structural homogeneity. The GROD (Grain Reference Orientation Deviation) distribution map was also determined while taking into account the possible precipitation of the secondary austenite phase and DRX process, depending on deformation temperature. The main conclusion is that the studied steel can be safely deformed by upsetting between 1050 °C and 1275 °C. Warm compression tests were carried out by Xu et al. [16] on low carbon and low alloy steel at temperatures of 600–850 °C. The evolution of microstructure and texture was studied using EBSD. The results indicated that cementite spheroidization greatly reduced at 750 °C due to a phase transformation. Dynamic recrystallization led to a transition from {112}<110> texture to {111}<112> texture. The contents of {111}<110> texture and {111}<112> texture were equivalent above 800 °C, resulting in the better uniformity of the γ-fiber texture. A temperature of 800 °C is suitable for the warm forming application, where the investigated material is easy to deform and evolves into a uniform and refined microstructure.

The following works [17,18] are related to the study of formability. The research of Du et al. [17] aimed to investigate the formability of Al–Si alloys reinforced with different fractions and different sizes of SiC particles to create an efficient and lightweight composite brake disk. Lanthanum and cerium were added to strengthen the aluminum matrix alloy and to improve the capability of the brake discs to withstand elevated temperature conditions, such as more extended braking periods. These elements formed intermetallic phases that further strengthened the composite. The additions of Ce and La strengthened the softer matrix regions and resulted in a doubled compression peak strength of the material without affecting the formability, as demonstrated by the processing maps. Rodak, Kuc, and Mikuszewski [18] studied superplastic deformation of Al–Cu alloys after grain refinement by extrusion combined with reversible torsion (KoBo). The binary as-cast Al–Cu alloys Al-5%Cu, Al-25%Cu, and Al-33%Cu (in wt%), composed of the intermetallic θ-Al2Cu and α-Al phases, were severely plastically deformed using extrusion coefficients of up to 98. KoBo enabled large elongation for alloys Al-25%Cu and Al-33%Cu (with higher intermetallic phase values) in the range from 830% to 1100% to be obtained by tensile testing at a temperature of 400 °C and a strain rate of 10^−4^ s^−1^. The degree of elongation depended on the extrusion coefficient and increased, as a result of α-Al and θ-Al2Cu phase refinement, to about 200–400 nm. A microstructural study showed that the mechanism of grain boundary sliding was responsible for superplastic deformation.

Several authors have dealt with both theoretical and technological aspects of forging. Banaszek et al. [19] investigated the influence of open-die forging parameters on the flow kinetics of AZ91 magnesium alloy. The paper presents the results of numerical simulations (using commercial Forge^®^NxT software) of the process of forging ingots on a hydraulic press with the use of flat and proprietary shaped anvils. The aim of this research was to reduce the number of forging passes. Analysis of the hydrostatic pressure distribution and of the equivalent strain was carried out. This is one of the elements used for determining the ability of forging technology to obtain a semi-finished product from the AZ91 alloy with good strength properties. An analysis of the formability, structure, and properties of the AZ61 cast magnesium alloy using the example of a new forging process of aircraft mount forgings was presented by Dziubińska, Surdacki, and Majerski [20]. It was assumed that their production process would be based on drop forging on a die hammer. Two geometries of preforms, differing in forging degree, were used as the billet for the forging process. Using a cast preform positively affected the formability and flow kinematics during forging and reduced the number of operations necessary to obtain the correct product. Numerical analysis of the proposed new technology was carried out using the commercial software DEFORM 3D v.11. The results obtained from numerical tests confirmed the possibility of forming the forgings of aviation mounts from the AZ61 cast alloy with the proposed technology. They also allowed information to be obtained regarding the kinematics of the material flow during forming and process parameters, such as strain distribution, temperature, Cockcroft–Latham criterion, and forming energy. The proposed forging process was verified in industrial conditions. The article of Banaszek et al. [21] discusses the impact of hot forging elongation operations on the closure of metallurgical discontinuities, such as middle porosity in selected magnesium alloys, depending on the shape of the input used. Laboratory simulations and numerical modeling, using the Forge^®^NxT 2.1 program based on the finite element method, were carried out in order to bring about the closure of defects of metallurgical origin in deformed forging ingots. Optimal values of the main technological parameters of forging and appropriate groups of anvils to be used in the individual stages of forging were proposed in order to eliminate the metallurgical defects. Using ultrasonic waves, Moravec, Bury, and Černobila [22] investigated selected characteristics of forging steel specimens for various levels of their relative reduction. The experimental procedure, using both the attenuation and velocity measurements, verified that the reduction in specimens’ material had an effect on the propagation of ultrasound waves passing through the body of the specimen. The increase in toughness after a relative reduction in forging in the range of 10–50% is, with highest probability, caused by the relatively important deformation hardening. The experiments were supplemented by Barkhausen’s noise detection and metallographic analysis of the specimens.

The paper by Bajor et al. [23] presents an analysis of the results of numerical simulations of the extrusion process of structural panels made from the 5xxx and 6xxx series aluminum alloys. The obtained products are intended for innovative superstructures of special car bodies. The main purpose of the research was to design a split die and to determine the parameters of the extrusion process. The distribution of stress, strain, strain rate, and temperature in the extruded metal was analyzed for two different punch movement speeds. It was shown that panel sections can be produced from ingots with a length of 770 mm on a press with the force of 35 MN. Mittelman et al. [24] studied the possibility to produce composite components by joining using plastic deformation, which results in metallurgical bonding at the interface. Beams of 6061 aluminum alloy were bonded by hot compression at temperatures of 300–500 °C to different degrees of reduction. The compression was followed by tensile debonding experiments, and the revealed interface was microscopically characterized in order to determine the areas that were metallurgically bonded. The actual bonded area was much smaller than the interface contact area. Thermo-mechanical finite element models of the compression forming were used to investigate the thermo-mechanical fields, which developed along the interface and influenced the resulting bonding strength. A quantitative criterion for bonding quality was implemented and shown to correlate with the experimental findings. 
